# Supplementary material for: Validation of a stereological method for estimating particle size and density from 2D projections with high accuracy
Source: PLoS One. 2023 Mar 17;18(3):e0277148. doi: 10.1371/journal.pone.0277148 (PMC10022809; doi:10.1371/journal.pone.0277148)
Supplement: S1 File — (PDF) [file pone.0277148.s001.pdf]

SUPPORTING INFORMATION TO:

Validation of a stereological method for estimating particle size and density from 2D projections with high accuracy

Jason Seth Rothman[1*], Carolina Borges-Merjane[2,#a], Noemi Holderith[3], Peter Jonas[2], R. Angus Silver[1*]

[1]Department of Neuroscience, Physiology and Pharmacology, University College London, London, United Kingdom.

[2]Cellular Neuroscience, Institute of Science and Technology Austria, Am Campus 1, 3400 Klosterneuburg, Austria.

[3]Laboratory of Cellular Neurophysiology, Institute of Experimental Medicine, Budapest 1083, Hungary.

[#a]Current address: Biozentrum of the University of Basel, 4056 Basel, Switzerland.

*Corresponding authors:

j.rothman@ucl.ac.uk (JSR)

a.silver@ucl.ac.uk (RAS)

Contains:

Appendix S1 and S2

Figure S1–S19

Appendix S1. Derivation of Eq 3 and why d_{min} is not a good measure of lost caps.

Abercrombie (1946) derived the following relation between the expected true particle count (N_{true}) and actual crude particle count ($N_{measured}$) of a section of thickness T, also known as the Abercrombie correction formula:

$$\frac{N_{true}}{T} = \frac{N_{measured}}{T+\mu_D} \qquad \text{Eq A1.1a}$$

Dividing both sides of this equation by the cross-sectional area over which the particles are counted ($Area_{xy}$) gives:

$$\frac{N_{true}}{Area_{xy}\cdot T} = \frac{N_{measured}}{Area_{xy}\cdot(T+\mu_D)} \qquad \text{Eq A1.1b}$$

Substituting in terms λ_{3D} and λ_{2D} used in this study gives:

$$\lambda_{3D} = \frac{\lambda_{2D}}{(T+\mu_D)} \qquad \text{Eq A1.1c}$$

However, this relation is only correct if there are no lost caps. If there are lost caps, then μ_D must be scaled smaller. Using the Keiding fixed-ϕ model (1972), scaling of μ_D can be achieved via cosϕ (Fig 1) and λ_{3D} can be estimated via the following (Cruz-Orive, 1983):

$$\lambda_{3D} = \frac{\lambda_{2D}}{(T+\mu_D\cos\phi)} \qquad \text{Eq A1.2a}$$

$$\lambda_{3D} = \frac{\lambda_{2D}}{\zeta} \qquad \text{Eq A1.2b}$$

which is Eq 3 of this study. Floderus (1944) derived a similar expression with respect to the section penetration depth of the smallest observable cap (h_{min}):

$$\lambda_{3D} = \frac{\lambda_{2D}}{(T+D-2h_{min})} \qquad \text{Eq A1.3}$$

and this expression was later recast by Konigsmark (1970) as a function of d_{min}, the diameter of the smallest observable cap:

$$\lambda_{3D} = \frac{\lambda_{2D}}{\left(T+\left[D^2-d_{min}^2\right]^{1/2}\right)} \qquad \text{Eq A1.4}$$

using the trigonometric relation:

$$2h_{min} = D - \left[D^2 - d_{min}^2\right]^{1/2} \qquad \text{Eq A1.5}$$

For analysing images, Eq A1.4 has proved more useful than the original Floderus equation since d_{min} can be directly measured from the sample of measured 2D diameters. However, an underlying assumption of the Floderus/Konigsmark correction is that all particles are the same size. If there is a distribution of particle size, then each particle will have its own minimum observable diameter (δ_{min}) and d_{min} will be the minimum of all δ_{min} (Fig 6C1 and S8C1). Depending on the spread of the δ_{min} distribution, the difference between d_{min} and the mean δ_{min} can be large, in which case the d_{min} correction will create a large underestimation of λ_{3D}. Moreover, there is a high probability that d_{min} is an outlier, i.e. an unusually small value; this could happen when conditions for identifying small caps are better than average, or when there are false positive measurements.

Our 3D analysis of MFT vesicles showed the Keiding model accurately describes the δ_{min}-D relation of the vesicles (Fig 6C1 and S8C1) and therefore gives an accurate estimate of λ_{3D} via Eq 3 (Fig 11). This is in contrast to the d_{min} correction which underestimated λ_{3D} for the same datasets by 13–20% and the Abercrombie correction which underestimated λ_{3D} by 22–24% (Table 5; ET10 and ET11). However, under ideal conditions where there are few lost caps, differences between λ_{3D} estimated via the Keiding model and the d_{min} and Abercrombie corrections are expected to be small. For example, our analysis of GC nuclei in planar sections, where $\phi = 20°$, showed only a 2–6% difference between estimated λ_{3D}.

Appendix S2. Estimation of the volume fraction of spherical particles from the area fraction of their 2D projections.

The relation between the volume fraction (VF) of spherical particles and their observed area fraction (AF) in a 2D projection was derived by Weibel and Paumgartner (1978; their Eq 13 and 37) and is as follows:

$$VF = K_v \cdot AF \qquad \text{Eq A2.1}$$

where

$$K_v = \frac{2m_3}{(2m_3 + 3g \cdot m_2 - 3X^2 + X^3)}$$

$$m_2 = \frac{(\mu_D^2 + \sigma_D^2)}{\mu_D^2}$$

$$m_3 = \frac{\mu_D \cdot (\mu_D^2 + 3\sigma_D^2)}{\mu_D^3}$$

$$g = \frac{T}{\mu_D}$$

$$X = \frac{2h_{min}}{\mu_D}$$

Here, m_2 and m_3 are dimensionless moments for a Gaussian distribution and $\mu_D = 2\mu_R$. Because ϕ is a better descriptor of the lost-cap distribution than h_{min} or its equivalent d_{min} (Appendix S1; Fig 6C1 and S8C1), we express X as a function of ϕ rather than h_{min} by first defining X with respect to d_{min} via Eq A1.5:

$$X = \frac{2h_{min}}{\mu_D} = \frac{\left(\mu_D - \left[\mu_D^2 - {d_{min}}^2\right]^{1/2}\right)}{\mu_D} \qquad \text{Eq A2.2a}$$

$$X = 1 - \left[1 - \left(\frac{d_{min}}{\mu_D}\right)^2\right]^{1/2} \qquad \text{Eq A2.2b}$$

and substituting $d_\phi = \mu_D \cdot \sin\phi$ for d_{min}:

$$X = 1 - cos\phi \qquad \text{Eq A2.2c}$$

To test this relation, we can consider the ideal scenario of a planar section with no lost caps (ϕ = 0°), in which case $g = 0$, X = 0, $K_v = 1$ and VF = AF, which is expected (Weibel

and Paumgartner, 1978). We can also consider the scenario where all particles have the same diameter (D), in which case $\sigma_D = 0$, $m_2 = m_3 = 1$ and:

$$K_v = \frac{2}{[2+3g-3X^2+X^3]} \quad \text{Eq A2.3a}$$

$$K_v = \frac{2}{[3g+3cos\phi-cos^3\phi]} \quad \text{Eq A2.3b}$$

$$K_v = \frac{2}{[3g+cos\phi\cdot(2+sin^2\phi)]} \quad \text{Eq A2.3c}$$

which is equivalent to Eq 31 of Weibel and Paumgartner if one substitutes d_ϕ for d_{min} (i.e. $d_{min}/D \rightarrow d_\phi/D = \sin\phi$). Finally, we computed the VF of the Monte Carlo simulations in Fig 4C using Eq A2.1 and A2.2c and found close agreement to the true VF (Fig 10C). Likewise, the VF computed via the same equations for our ET z-stack analysis showed close agreement to the VF computed via our 3D analysis (Fig 11; Table 2).

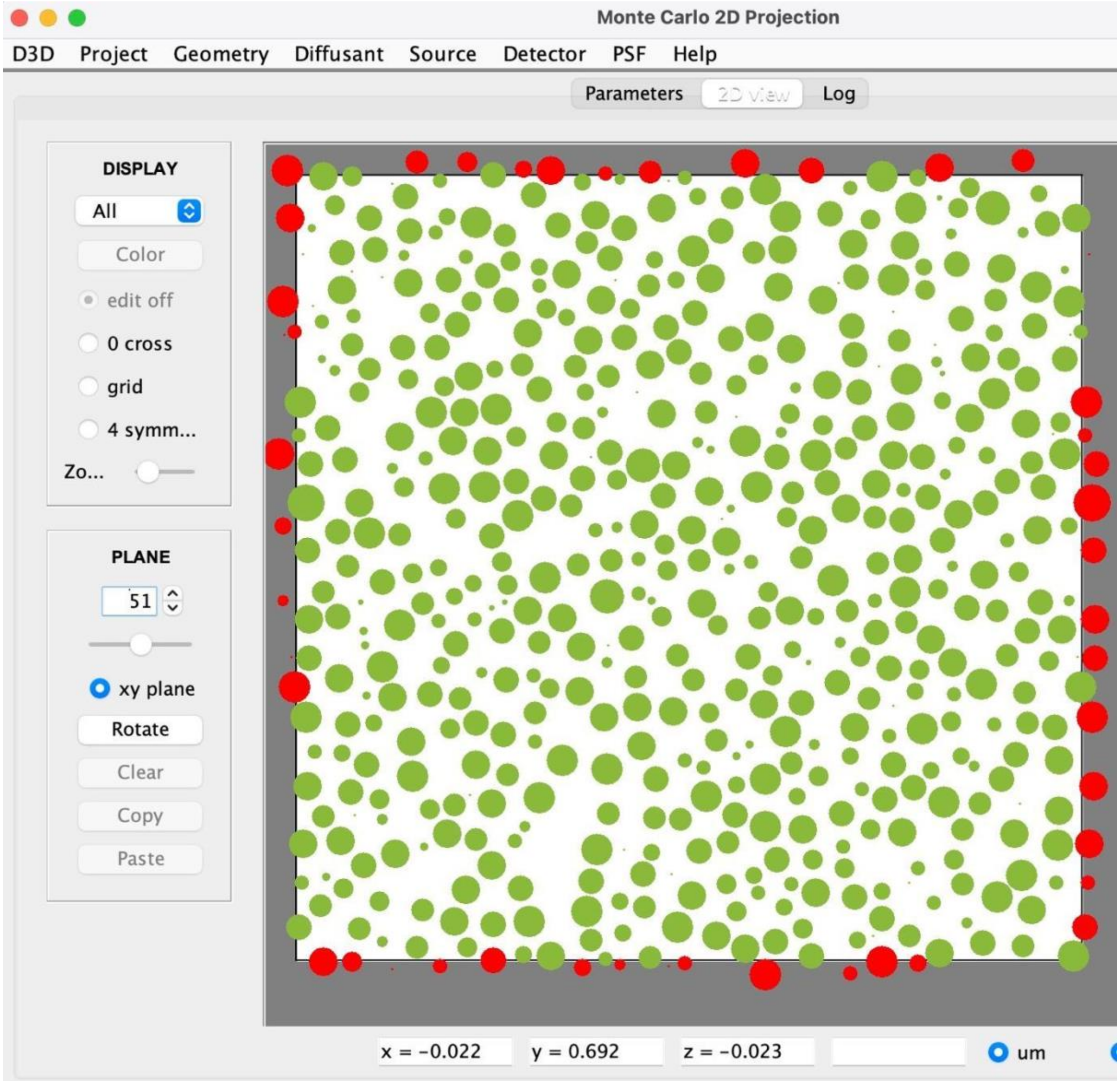

Fig S1. Monte Carlo simulation of a 2D projection of spherical particles.

2D projection of a planar section (T = 0, ϕ = 40°) through the middle of a Monte Carlo simulation (Materials and Methods) in which spherical particles were randomly distributed in a cuboid geometry (26 × 26 × 104 u.d.) where VF = 0.40. F(d) was a Gaussian distribution with normalised mean (Eq 5; $\mu_D \pm \sigma_D$ = 1.00 ± 0.09 u.d.). Red circles denote particles reflected at the x and y borders, i.e. periodic boundary conditions, shown only for display purposes (i.e. they do not contribute to any size or density analysis). The geometry z-dimension was deep enough to accommodate 100 such projections. The simulation was computed via D3D (https://github.com/SilverLabUCL/D3D).

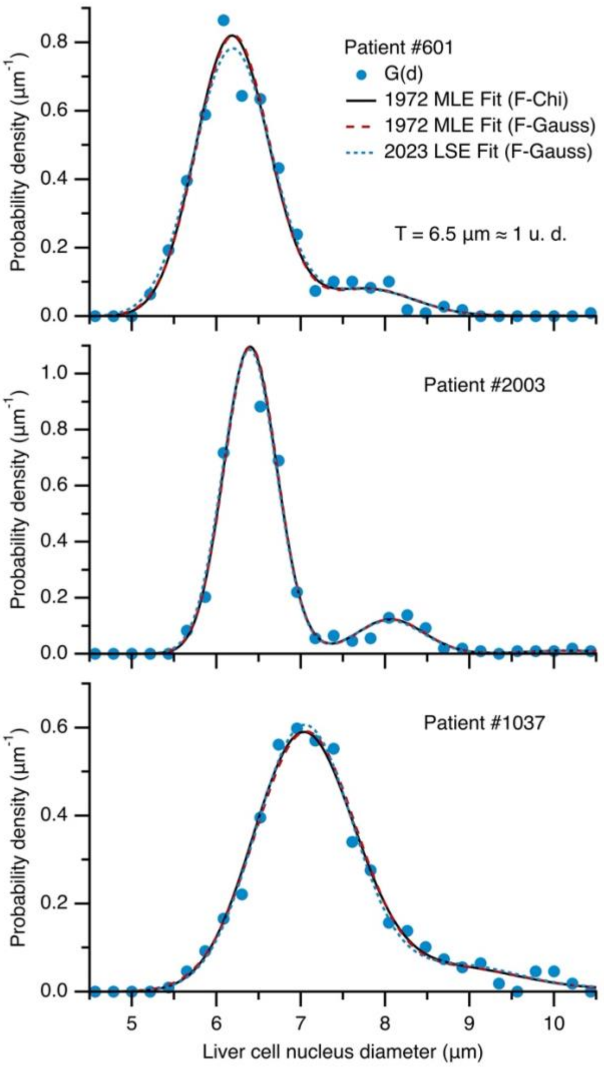

Fig S2. Replication of the original Keiding-model fits to G(d) of liver cell nuclei. G(d) of liver cell nuclei (blue circles) computed from Table 1 of Keiding et al. (1972) for patients 601, 2003 and 1037. Distribution x-axes radii (units of mm) were converted to diameters (units of µm) via the following conversion factor: $\Theta = 2\times1000/2300$ µm/mm, where 2300 corrects for magnification. The maximum likelihood estimation (MLE) fits of Keiding et al. (black lines) were computed by plugging the parameters from their Table 2 (f, β, ϕ, p1, p2) into a modified version of Eq 1 (NMKeidingChi3) that includes the weighted sum of 3 G(d) as described by Keiding et al. (their Eq 4.1) using a chi distribution for F(d) (K-Chi3; Eq 6) and converting β from units of square radii (mm^2) to square diameters (μm^2) by multiplying by Θ^2. For comparison, MLE fits were recomputed using a Gaussian distribution for F(d) (red lines; K-Gauss3; NMKeidingGauss3; Eq 5) where μ_D and σ_D were computed from f and β. The overlap of these two curves (K-Chi3 vs. K-Gauss3) demonstrates the estimated chi distributions from the MLE fits are approximately Gaussian, which is expected since f is large for these fits (104, 208 and 70). As a last comparison, the same modified version of Eq 1 (NMKeidingGauss3) was curve fitted to the 3 G(d) using our LSE routine (blue dashed lines) resulting in nearly equivalent curves and parameters (Table 4).

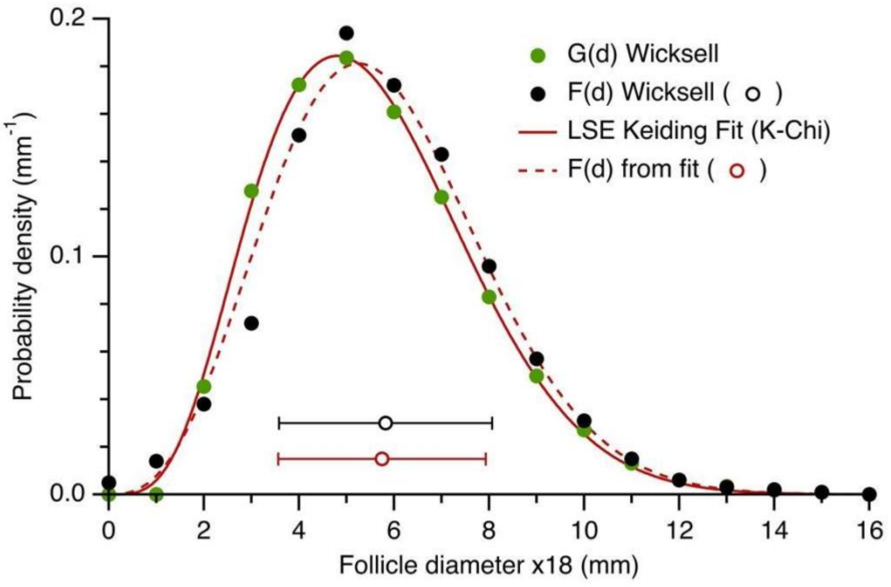

Fig S3. Keiding-model curve fit to G(d) of Wicksell's corpuscle problem.

Curve fit of Eq 1 (red solid line) to G(d) of Wicksell's corpuscle problem (1925; green circles) where F(d) was defined by the chi distribution (K-Chi; Eq 6). F(d) derived from the fit (red dashed line and circle; $\mu_D \pm \sigma_D$ = 5.75 ± 2.18 mm) matches Wicksell's F(d) computed via a finite-difference unfolding algorithm (black circles; $\mu_D \pm \sigma_D$ = 5.82 ± 2.24 mm). Keiding-model fit f = 3.67 ± 0.33, β = 10.31 ± 0.61 mm^2, ϕ = 25 ± 3°, χ^2 = 0.0001. X-scale of Wicksell includes 18-fold magnification. T = 0.018 mm. Note, assuming a Gaussian function for F(d) (K-Gauss; Eq 5) resulted in a poor fit (χ^2 = 0.002).

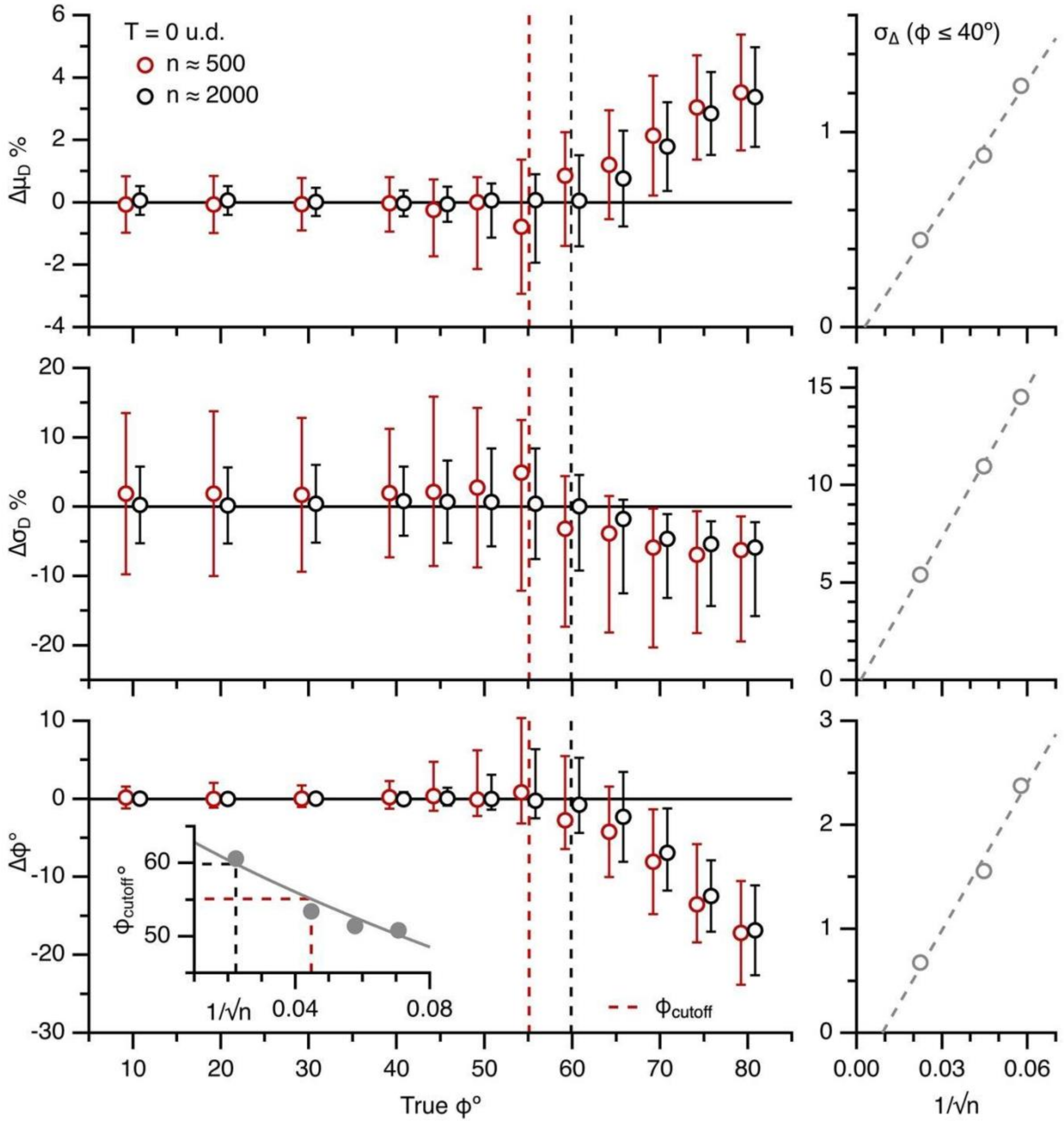

Fig S4. The Keiding model accurately estimates F(d) and ϕ from G(d) for true ϕ < ϕ_{cutoff} (500 vs. 2000 simulated diameters).

Left column: average $\Delta\mu_D$, $\Delta\sigma_D$ and $\Delta\phi$ of Keiding-model fits to simulated G(d) computed from ~500 diameters (red circles; data from Fig 4C) and ~2000 diameters (black circles) for true ϕ = 10–80°, T = 0 u.d., CV_D = 0.09. Red and black dashed lines denote respective ϕ_{cutoff} (~55 and 60°; Eq 8). Data x-scales shifted ±0.8° to avoid overlap. Bottom inset: ϕ_{cutoff} vs. 1/√n for simulations (gray circles) and Eq 8 (gray line; CV_D = 0.09). Right column: 68% confidence interval (σ_Δ) of $\Delta\mu_D$, $\Delta\sigma_D$ and $\Delta\phi$ (averaged across true ϕ = 10–40°) vs. 1/√n (n = 300, 500 and 2000 diameters) curve fitted to a linear function (dashed lines).

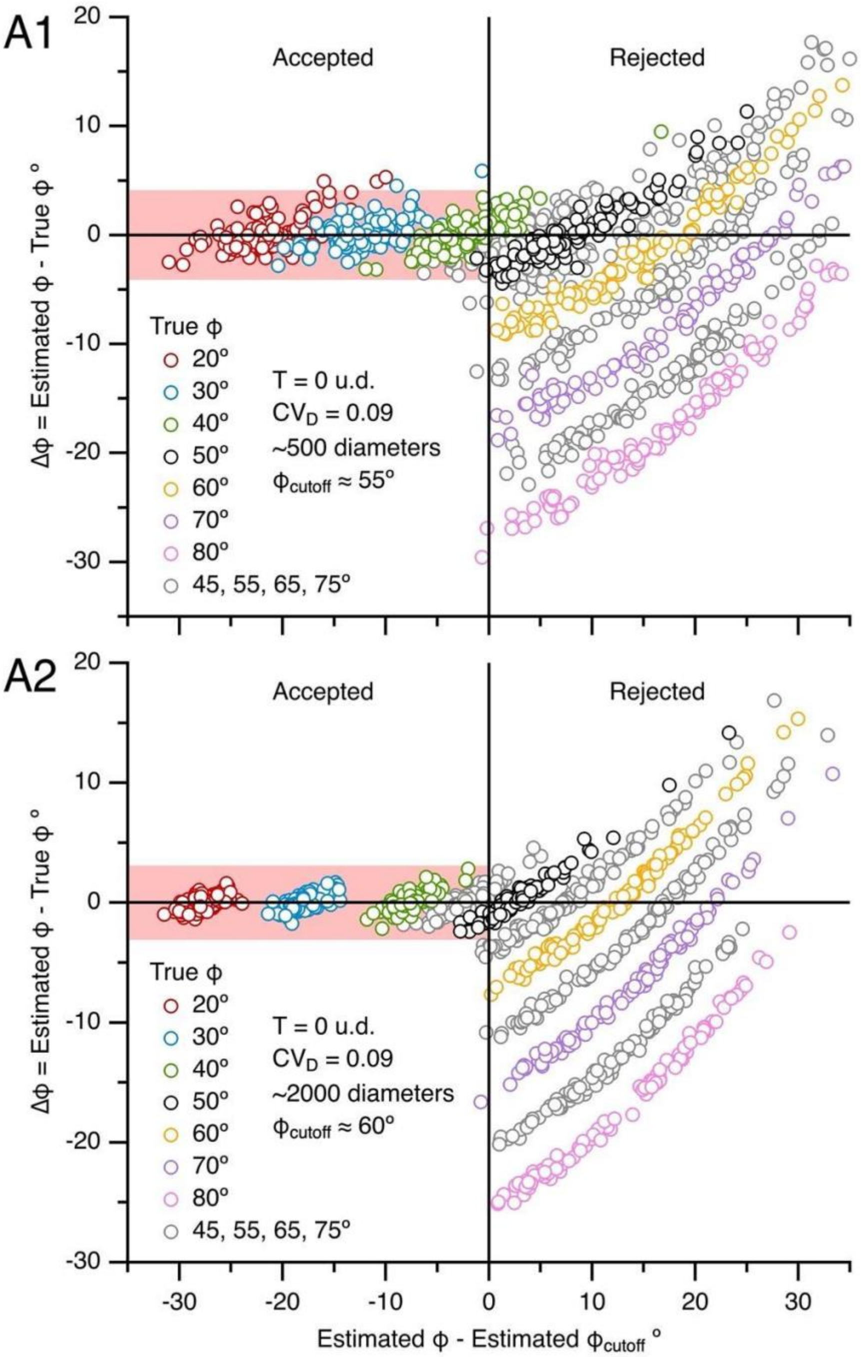

Fig S5. The ϕ-accuracy test using estimated ϕ_{cutoff}.

Δϕ of Keiding-model fits to simulated G(d) versus the difference between estimated ϕ and estimated ϕ_{cutoff}, where estimated ϕ_{cutoff} was computed via Eq 9 using estimated μ_D and σ_D. These plots show that if estimated ϕ < estimated ϕ_{cutoff} (left side of graphs) there is a high probability |Δϕ| < 4° for G(d) computed from ~500 diameters (**A1**; red shading; data from Fig 4C), |Δϕ| < 3° for G(d) computed from ~2000 diameters (**A2**; data from Fig S4) and |Δϕ| < 5° for G(d) computed from ~300 diameters (not shown). For the simulations, true ϕ = 20–80°, T = 0 u.d., CV_D = 0.09, with 100 simulated G(d) per true ϕ. A few data points are off scale.

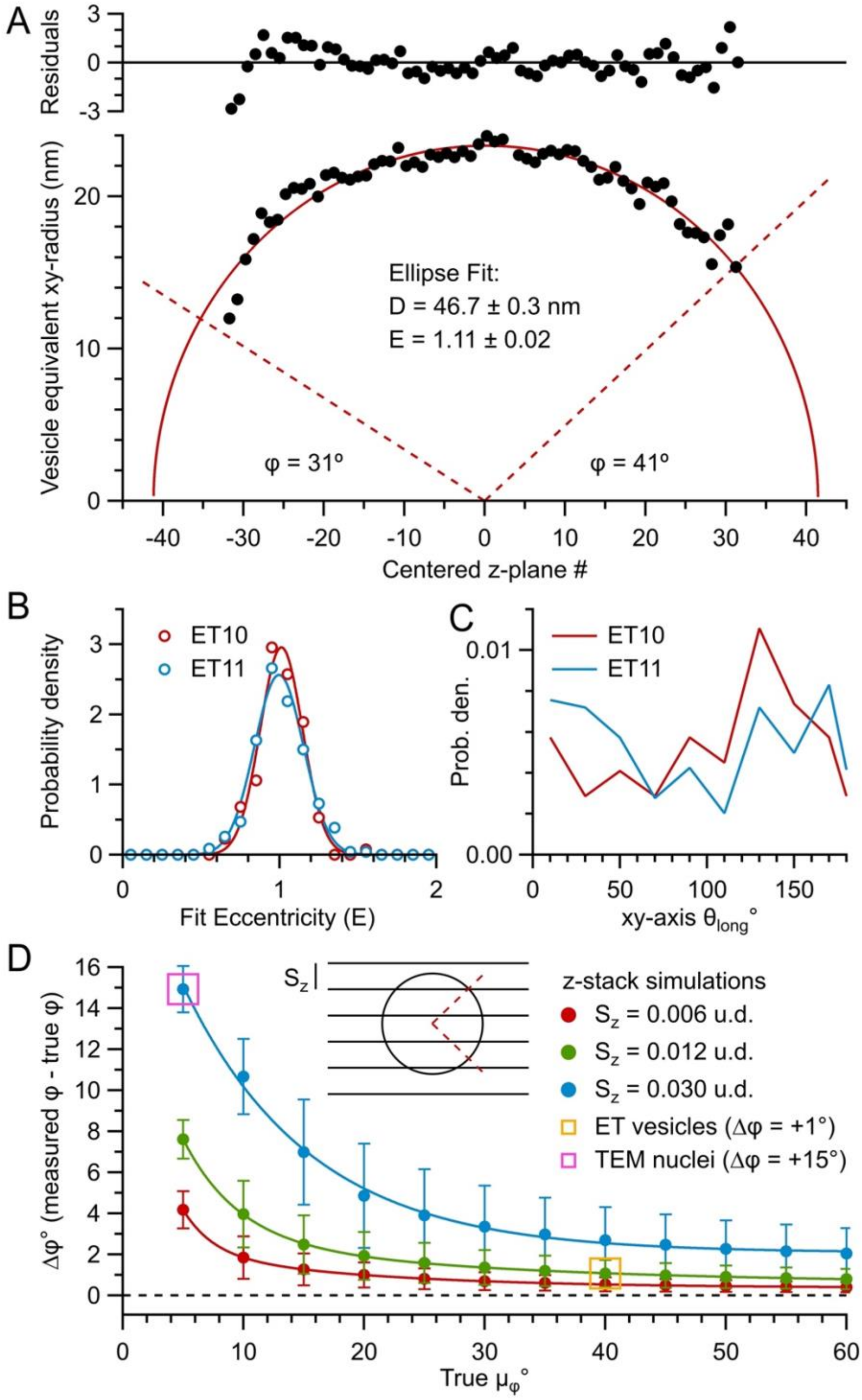

Fig S6. 3D analysis of MFT vesicles in ET z-stacks.

A. Example curve fit of an ellipse (Eq 10; red solid line) to the equivalent xy-radius (½d_{area}) vs. z-stack image number ($z_{\#}$) relation of a MFT vesicle (black circles; vesicle #20) from z-stack ET10 where the z-axis was centered at $z_{\#} = 0$. Top graph shows residuals (nm) between data and fit. Red dashed lines denote measured ϕ for the vesicle's north and south poles computed as $\phi = \sin^{-1}(\delta_{min}/D)$, where δ_{min} is the minimum diameter measured at the given pole.

B. Probability density of eccentricity factor (E; 0.1 bins) for curve fits of Eq 10 to the xy-radius vs. $z_{\#}$ relations, as in **A**, of vesicles in ET10 (red; n = 132) and ET11 (blue; n = 233). For ET10 and ET11, S_z was fixed to 0.63 and 0.53 nm, respectively, the values necessary to obtain an average E = 1.00, i.e. an average z-axis diameter equal to the average xy-axis diameter (isotropic conditions). These S_z are 1.7- and 1.4-fold larger than the acquisition S_z (0.38 nm) indicating the sections after imaging were 60% and 70% of their original thickness, a shrinkage that is consistent with previous findings (Luther et al. 1988). The narrow E distributions (σ = ±0.15 and ±0.16) indicate spherical dimensions. See Fig S7, S8D and 6D for corresponding plots of parameter D, i.e. F(d).

C. Probability density (per °) of θ_{long} (orientation of longest vesicle diameter; 20° bins) for ET10 (red; n = 122) and ET11 (blue; n = 271) showing the vesicles have no systematic orientation in the xy-plane, consistent with a random orientation. For a given vesicle, θ_{long} was measured on the z-plane where the vesicle has a maximum d_{area}.

D. Discretization error of measured ϕ (Δϕ = measured ϕ – true ϕ) as a function of z-stack z-resolution (S_z) for Monte Carlo simulations where true CV_ϕ = 0.2 and VF = 0.35. Data is the average of 100 sections ($\mu_\Delta \pm \sigma_\Delta$). Orange square denotes conditions for the ET z-stacks of MFT vesicles (Fig 6 and S8; nonblind analysis; assuming true ϕ = 40°). Pink square denotes conditions for TEM z-stack of GC nuclei (Fig S9; nonblind analysis; assuming true ϕ = 5°). Inset: cartoon of a particle sectioned within a z-stack. Dashed red lines denote the particle's ϕ.

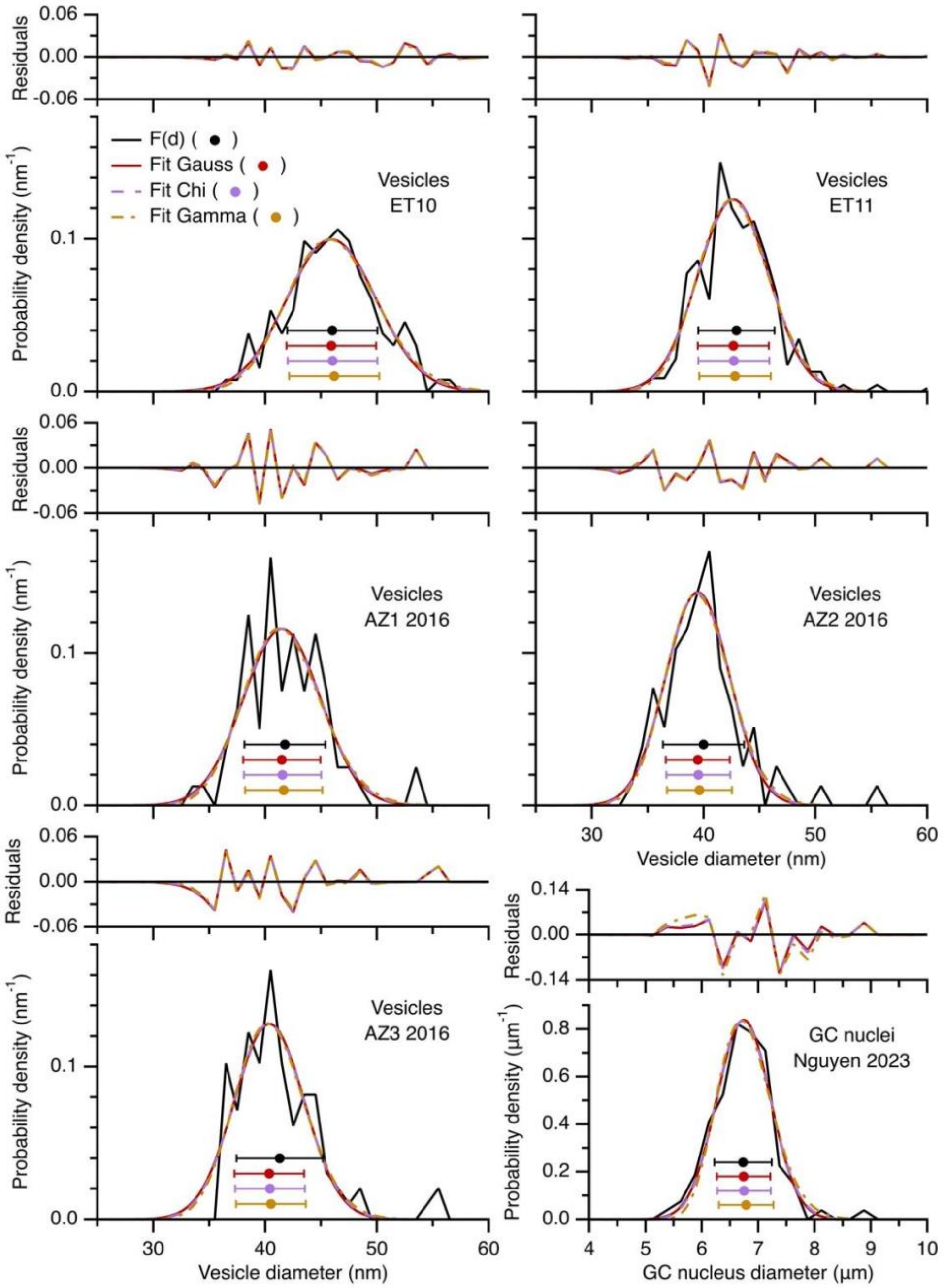

Fig S7. F(d) of cerebellar MFT vesicles and GC nuclei is well described by a Gaussian distribution.

Comparison of LSE curve fits of a Gaussian distribution (Eq 5; red line), chi distribution (Eq 6; purple line) and gamma distribution (Eq 7; yellow line) to F(d) (black line) in Fig S8D (ET10; n = 132 diameters) and 6D (ET11; n = 233 diameters) and three F(d) from a previous study (Rothman et al. 2016; AZ1–3; n = 80, 78, 98 diameters) all computed from ET z-stacks of MFT vesicles. The fits overlap and show little difference in residuals (nm), χ^2 and $\mu_D \pm \sigma_D$ (circles ± error bars). For fits to the gamma distribution, d_0 was fixed at 10 nm, a value determined by an initial round of fits where d_0 was allowed to vary. Similar results were found for F(d) of GC nuclei measured from a TEM z-stack (Fig S9D; Nguyen et al. 2023; gamma d_0 fixed at 4 μm).

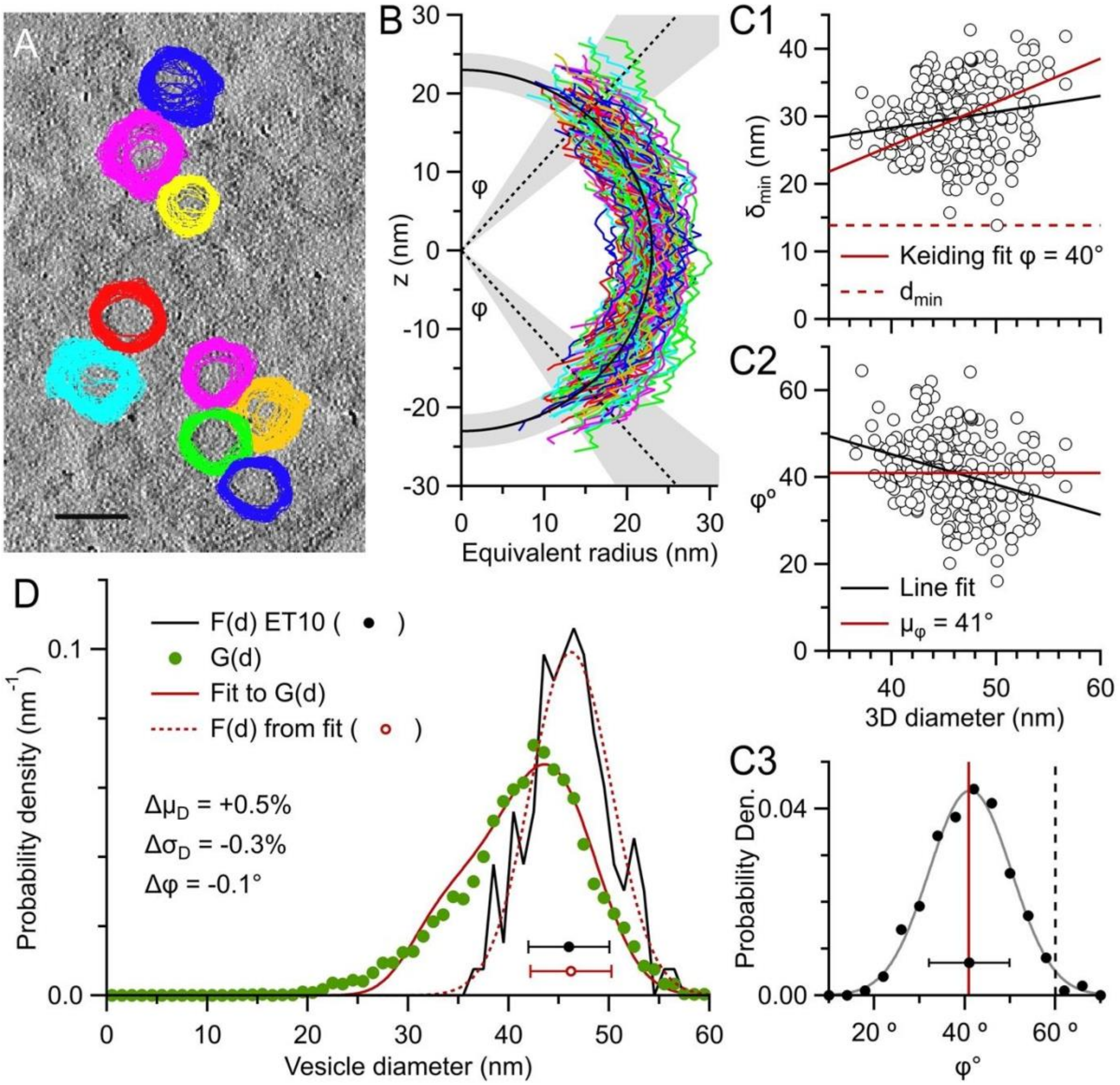

Fig S8. The Keiding model accurately estimates F(d) and ϕ from G(d) for true ϕ < ϕ_{cutoff} (vesicles ET10).

Same as Fig 6 for ET10.

A. One of 291 serial images of a 3D ET reconstruction (ET10) of a cerebellar MFT section 182 nm thick. 141 vesicles, including 24 caps, were tracked and outlined through multiple z-planes (i.e. a nonblind particle detection) and their d_{area} computed as a function of z-plane number. This image shows outlines for 9 representative vesicles, overlaid with outlines from images above and below. Scale bar 50 nm.

B. Black semi-circle and shading denote F(d) = $\mu_D \pm \sigma_D$ = 46.0 ± 4.0 nm for all measured 3D diameters (D; n = 132). Black dotted lines and shading denote measured ϕ: $\mu_\phi \pm \sigma_\phi$ = 41 ± 9° (CV_ϕ =0.2; n = 249, measures of north and south poles, including caps). Fit E = 1.00 ± 0.15 (n = 132). Fits to the smallest caps were not included (n = 9).

C1. δ_{min} vs. D (circles; n = 249) with line fit (black line; χ^2 = 6583, r = 0.2, R^2 = 0.03) and Keiding-model fit (red solid line; fit ϕ = 39.9 ± 0.4°; χ^2 = 7233, r = 0.2, R^2 = 0.2).

C2. ϕ vs. D for data in **C1** with line fit (black line; r = -0.3, R^2 = 0.1) and μ_ϕ = 41° (red solid line). $\phi = \sin^{-1}(\delta_{min}/D)$.

C3. Probability density (per °) of measured ϕ in **C2.** with Gaussian fit (gray line; Eq 5) and fit ϕ (red line) and ϕ_{cutoff} from **D** (black dashed line; ~60°; Eq 8).

D. Measured F(d) (black line and circle; see **B**) vs. G(d) (green circles; n = 7083 outlines). A curve fit of Eq 1 to G(d) (red solid line; μ_D = 46.2 ± 0.1 nm, σ_D = 4.0 ± 0.1 nm, ϕ = 41 ± 1°; T fixed to 0 nm) resulted in estimated F(d) (red dotted line and circle) nearly the same as measured F(d) and estimated ϕ the same as μ_ϕ (**C3**).

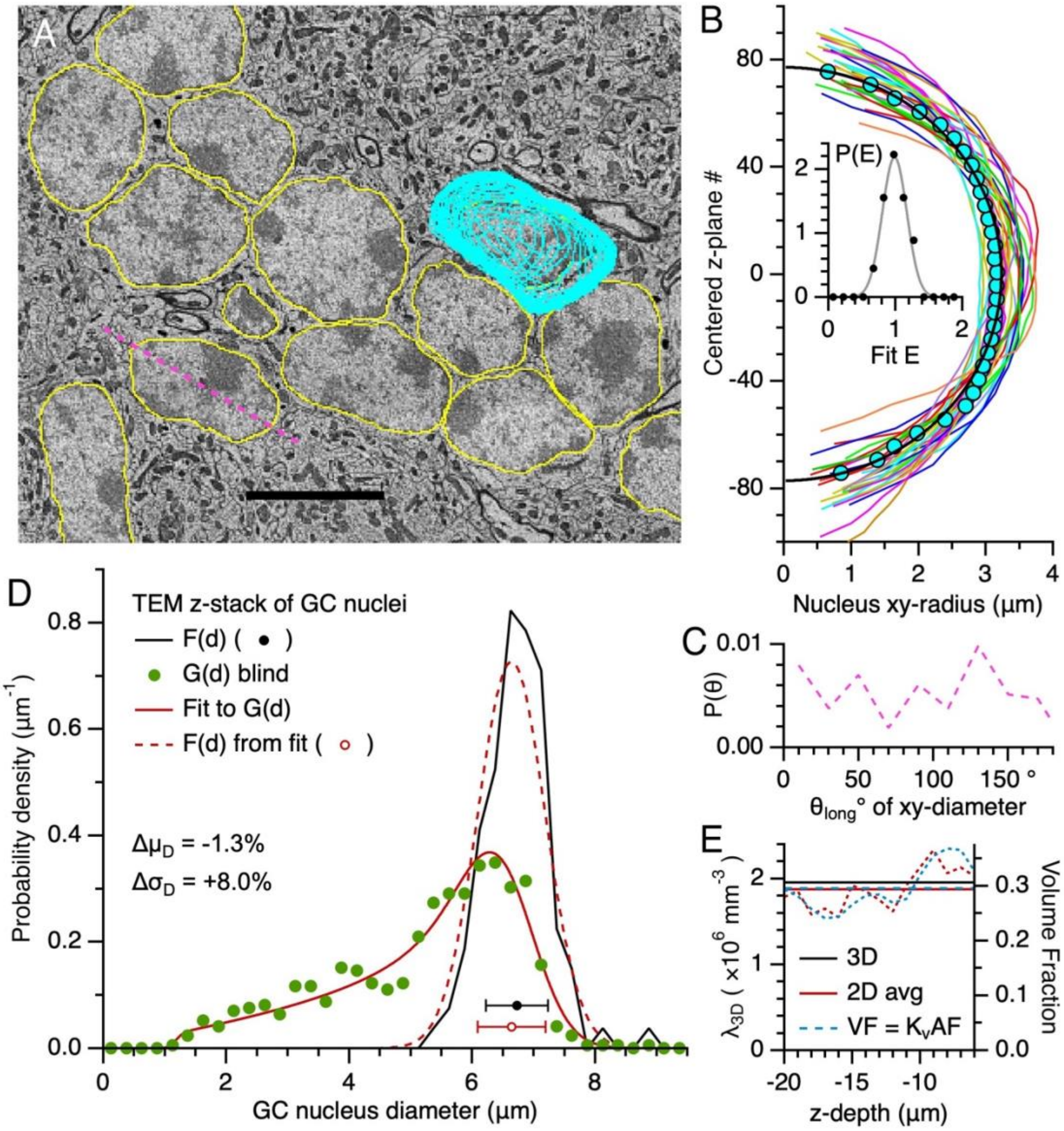

Fig S9. Size and density analysis of cerebellar GC nuclei computed from a TEM z-stack.

A. TEM image of a cerebellar section of a WT mouse (P40) from the online z-stack of Nguyen et al. (2023; see Data Availability; z-stack plane $z_{\#}$ = 150). Outlines were drawn around the outer contour of visually identified GC nuclei (yellow; blind detection) and d_{area} computed from the outlines. A subset of nuclei (n = 30) were tracked through multiple z-planes in steps of 5 (0.2 μm) with one shown here (cyan; nonblind detection; overlaid outlines from images above and below). Pink dashed line illustrates a measurement of a nuclei's xy-orientation with respect to its long diameter (θ_{long} = 144°). Only a subregion of the analysis is shown. Scale bar 6 μm.

B. Equivalent xy-radius (r = ½d_{area}) vs. $z_{\#}$ relation of the 30 nuclei tracked through multiple z-planes (lines), including the nucleus in **A** (cyan circles). The r-$z_{\#}$ relations were curve fitted to an ellipse (Eq 10), with one fit shown here for the nucleus in **A** (black line; D = 6.47 ± 0.04 μm, E = 0.96 ± 0.01). For all fits, S_z was fixed to 40 nm, the value necessary to obtain an average E = 1.00, i.e. an average z-axis diameter

approximately equal to the average xy-axis diameter (isotropic conditions). This S_z is similar to that reported by Nguyen et al. (45 nm). Z-axes were centered at $z_\# = 0$. Inset: probability density of E (black circles; 0.15 bins) curve fitted to a Gaussian function (gray line; Eq. 5). The narrow E distribution ($\sigma = \pm 0.17$) indicates spherical dimensions. Measures of δ_{min} and ϕ are not reported due to a large discretization error for this dataset (Fig S6D).

C. Probability density (per °) of θ_{long}, illustrated in **A**, showing the GC nuclei have no systematic orientation in the xy-plane, consistent with a random orientation (n = 107; 20° bins). For a given nucleus, θ_{long} was measured on the z-plane where the nucleus has a maximum d_{area}, described in **D**.

D. G(d) (green circles; 0.25 µm bins; n = 688 outlines) computed from d_{area} as in **A** (blind detection) for 15 z-planes interior to the z-stack ($z_\#$ = 100–450 in steps of 25). A curve fit of Eq 1 (red solid line) to G(d) resulted in an estimate of F(d) (red dotted line and circle; $\mu_D = 6.64 \pm 0.03$ µm, $\sigma_D = 0.55 \pm 0.03$ µm) that matched the measured F(d) (black line and circle; $\mu_D \pm \sigma_D = 6.73 \pm 0.51$ µm; $\Delta\mu_D$ = -1.3% and $\Delta\sigma_D$ = +8.0%) with a ϕ (10 ± 2°) that was determinable ($\phi_{cutoff} \approx 45°$; Eq 9). Measured F(d) was computed by tracking GC nuclei (n = 107) through multiple z-planes and measuring d_{area} on the plane where the nuclei appeared to have a maximum cross-sectional area (i.e. max d_{area}); this method of estimating D is likely to have overestimated D in comparison to D computed via a curve fit to the r-$z_\#$ relation (**B**; $\Delta\mu_D$ = +1.7%; n = 30). Repeating the analysis for G(d) computed from the data in **B** (nonblind detection; n = 974 outlines) gave a similar estimated F(d) ($\mu_D = 6.63 \pm 0.02$ µm, $\sigma_D = 0.52 \pm 0.02$ µm; $\Delta\mu_D$ = -1.4% and $\Delta\sigma_D$ = +1.8%) but smaller ϕ (6 ± 1°; $\phi_{cutoff} \approx 48°$). The small difference in ϕ between the blind and nonblind analysis (ϕ_{bias} = 4°) indicates the blind nuclei detection was capable of identifying nearly all caps. Note, these estimates of μ_D are 1.4-fold larger than that estimated for our analysis of GC nuclei in TEM images (Table 3).

E. GC nucleus density computed via the 2D analysis: $\lambda_{3D} = \lambda_{2D} / \zeta = 1.88 \times 10^6$ mm^{-3} (solid red line; Eq 3) where λ_{2D} = 820 / (15 × 4429 µm^2) = 12.3 × 10^3 mm^{-2} (n = 15 TEM images from **D**; blind detection) and ζ = 6.58 µm (Eq 2); dotted red line shows λ_{3D} for the 15 z-planes as a function of distance from the z-stack center (VF = 0.29; Eq 4). This estimated λ_{3D} is similar to that computed via a 3D analysis (black line; λ_{3D} = N_{3D}/$Volume_{xyz}$ = 206/105,335 µm^3 = 1.96 × 10^6 mm^{-3}; VF = 0.31 via Eq 4) and the 3D reconstruction of Nguyen et al. (1.87 × 10^6 mm^{-3}; their Fig 2). Comparison of the 2D versus 3D estimates of VF showed similar results (2D: VF = K_V·AF = 0.29 where AF = 0.30 and K_V = 0.99; Eq A2.1 and B.2c). While estimates of λ_{3D} are 3-fold smaller than that for our analysis of GC nuclei in TEM images, estimates of VF are only 1.2-fold smaller (Table 3). Left and right axes are equivalent scales for $\mu_D \pm \sigma_D = 6.64 \pm 0.55$ µm.

Only a subset of the original TEM z-stack was analysed: coordinate pixels x = 124016–146544, y = 95976–108264, z = 70–495 in z-steps of 5; however, images for z = 135, 175 and 445 were non-existent in the online database and substituted with z = 136, 176 and 446. Images were down-sampled by a factor of 10, giving S_{xy} = 40 nm/pixel.

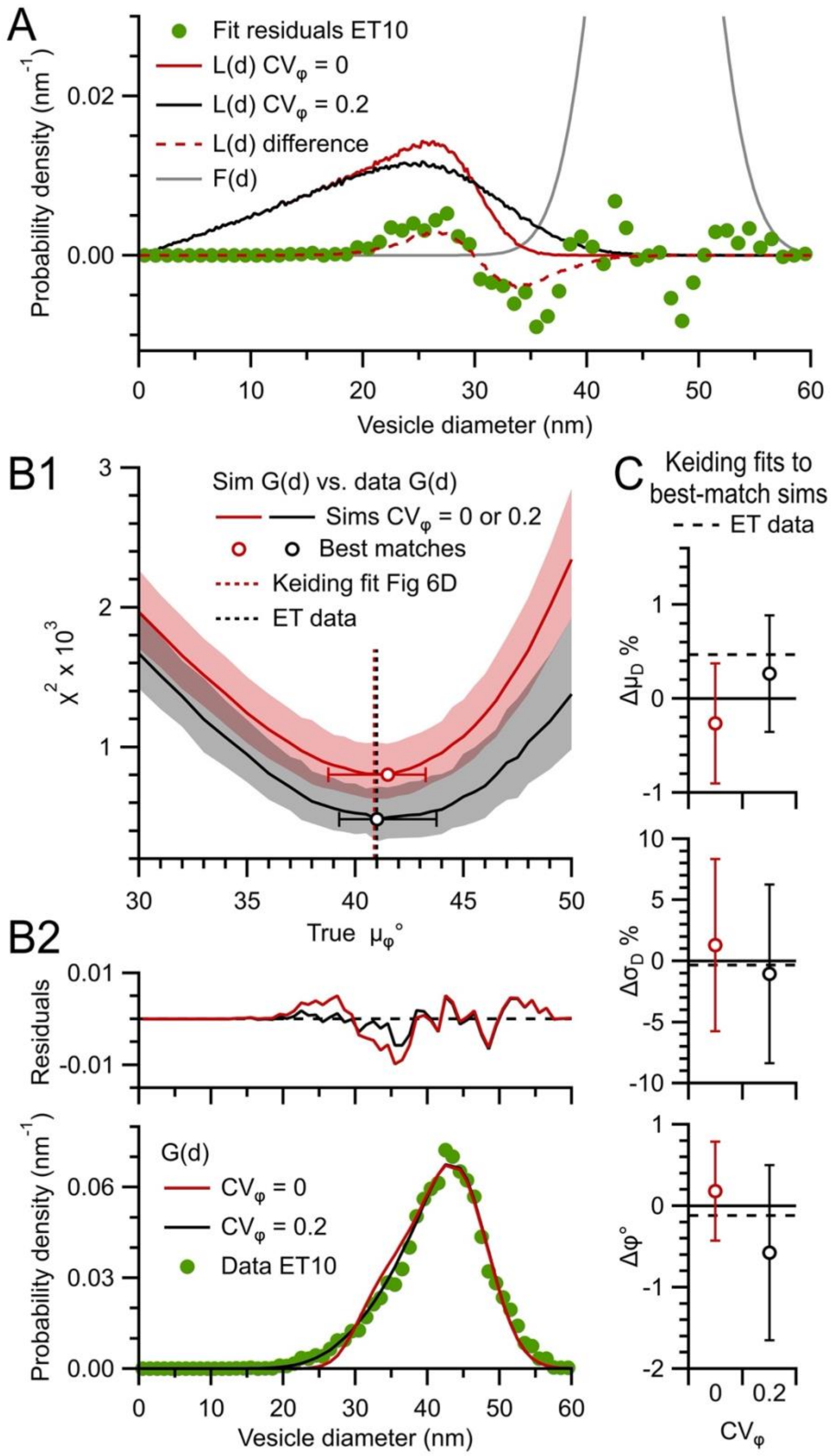

Fig S10. The Keiding-model fixed-ϕ assumption introduces only small errors when estimating F(d) and ϕ from G(d) (z-stack simulations).

A. Distribution of lost caps, L(d), for the Keiding-model fit to G(d) of ET10 (Fig S8D) where $\phi = 41°$ (red solid line; $CV_\phi = 0$; see Fig 3F) and Gaussian distribution $\phi = 41 \pm 9°$ (black solid line; $CV_\phi = 0.2$). The difference between these two distributions (red

dashed line) matches the residuals (nm) of the Keiding-model fit to G(d). Hence, the deviation of the Keiding-model fit from G(d) at 20–40 nm can be explained by the difference between a model that assumes a fixed ϕ (the Keiding model, 1972) and a Gaussian ϕ. L(d) was computed via simulations. F(d) is shown for comparison (gray line) and goes off scale.

B1. χ^2 comparison of experimental G(d) of ET10 to G(d) computed from a Monte Carlo model that assumes either a fixed ϕ as in the Keiding model ($CV_\phi = 0$; red solid line denotes 50% probability and shading denotes 16–84% probability computed from 100 repetitions per μ_ϕ) or a Gaussian ϕ that matches the experimental data ($CV_\phi = 0.2$; black solid line and shading). The Gaussian-ϕ model had significantly smaller χ^2 than the fixed-ϕ model. To simulate the z-stack data, F(d) of the simulated particles was matched to the measured F(d) ($\mu_D \pm \sigma_D = 46 \pm 4$ nm) and 2D diameters were computed from particle projections within an xy-plane (0.28 × 0.28 µm; T = 0 nm) that was z-shifted 290 times in 0.6 nm steps. The number of analysed particles (~140) and 2D diameters (~6600) was similar that of the experimental data for $\mu_\phi = 41°$. μ_ϕ was the only free parameter and was varied between 37–48° in steps of 0.5°, or 1° outside this region. Circles denote best-match μ_ϕ of the fixed-ϕ model (41.5°) and Gaussian-ϕ model (41.0°), as described below. Particle VF = 0.45. Red vertical dashed line denotes ϕ of the Keiding-model fit to experimental G(d). Black vertical dashed line denotes measured μ_ϕ. $\phi_{cutoff} \approx 60°$ (Eq 8).

B2. Experimental G(d) (bottom; green circles) compared to best-match simulated G(d) in **B1**, where simulated G(d) are the average for 100 repetitions for $\mu_\phi = 41°$ (red and black lines). Differences between experimental and simulated G(d) (top) shows the Gaussian-ϕ model is a better match to experimental G(d).

C. Average $\Delta\mu_D$, $\Delta\sigma_D$ and $\Delta\phi$ of curve fits of Eq 1 to the 100 G(d) of the best-match simulations in **B1**, computed with respect to 'measured' μ_D, σ_D and μ_ϕ of the particles in the projection. Black dashed lines denote $\Delta\mu_D$, $\Delta\sigma_D$ and $\Delta\phi$ of the experimental data. Errors of the Gaussian-ϕ model match the experimental errors better than those of the fix-ϕ model.

To find the best-match μ_ϕ for the given experimental G(d), the sum of squared differences (χ^2) was computed between the experimental G(d) and simulated G(d). Cumulative distribution functions (CDFs) of χ^2 were computed from the 100 simulated G(d) at a given μ_ϕ. From the CDFs, χ^2 values with 50% probability (χ^2-50%) were computed as a function of μ_ϕ, as well as χ^2 values with 16 and 84% probabilities representing $\pm\sigma$. The μ_ϕ with smallest χ^2-50% was deemed the best match. Confidence intervals above and below the best-match μ_ϕ denote the range of μ_ϕ whose χ^2 are not significantly different to that of the best-match μ_ϕ, computed via a KS test ($p > 0.05$).

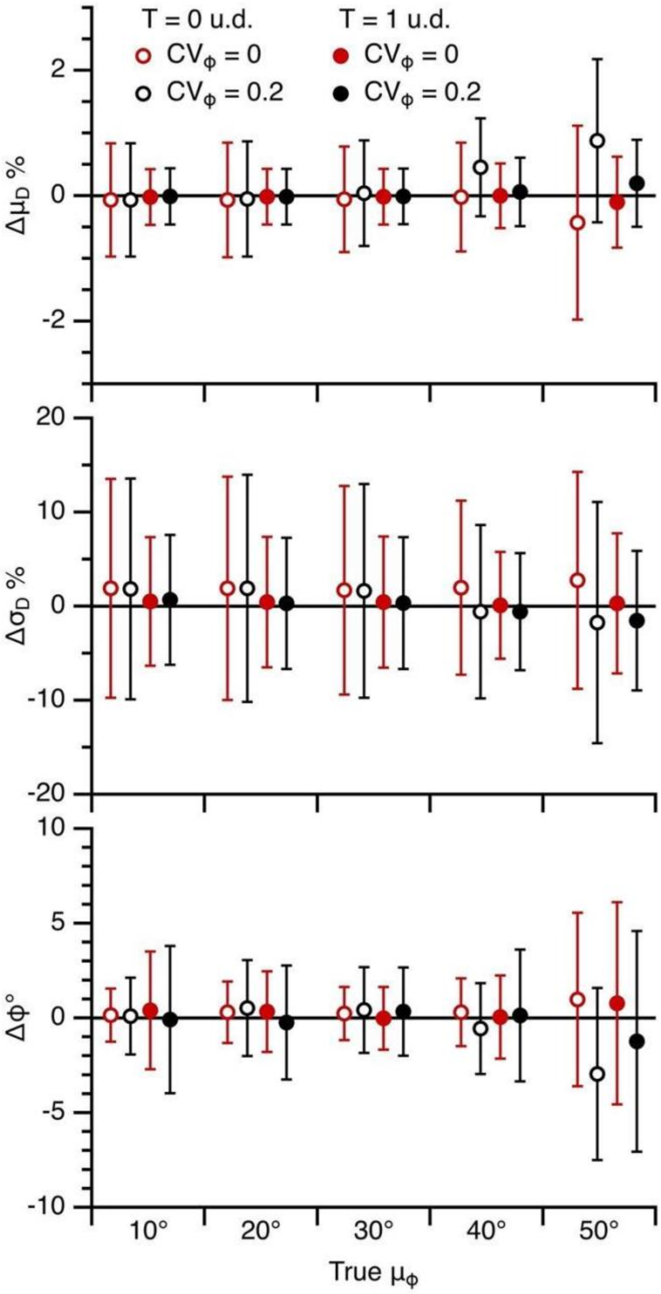

Fig S11. The Keiding-model fixed-ϕ assumption introduces only small errors when estimating F(d) and ϕ from G(d) (2D projection simulations).

Average $\Delta\mu_D$, $\Delta\sigma_D$ and $\Delta\phi$ of curve fits of Eq 1 to 100 G(d) computed from simulations as in Fig 4 for ~500 particles with a fixed or Gaussian ϕ for μ_ϕ = 10–50° (CV_ϕ = 0 or 0.2; red vs. black) and T = 0 and 1 u.d. (open and closed circles). Fixed-ϕ simulation data is from Fig 4C. ϕ_{cutoff} ≈ 55° (Eq 8).

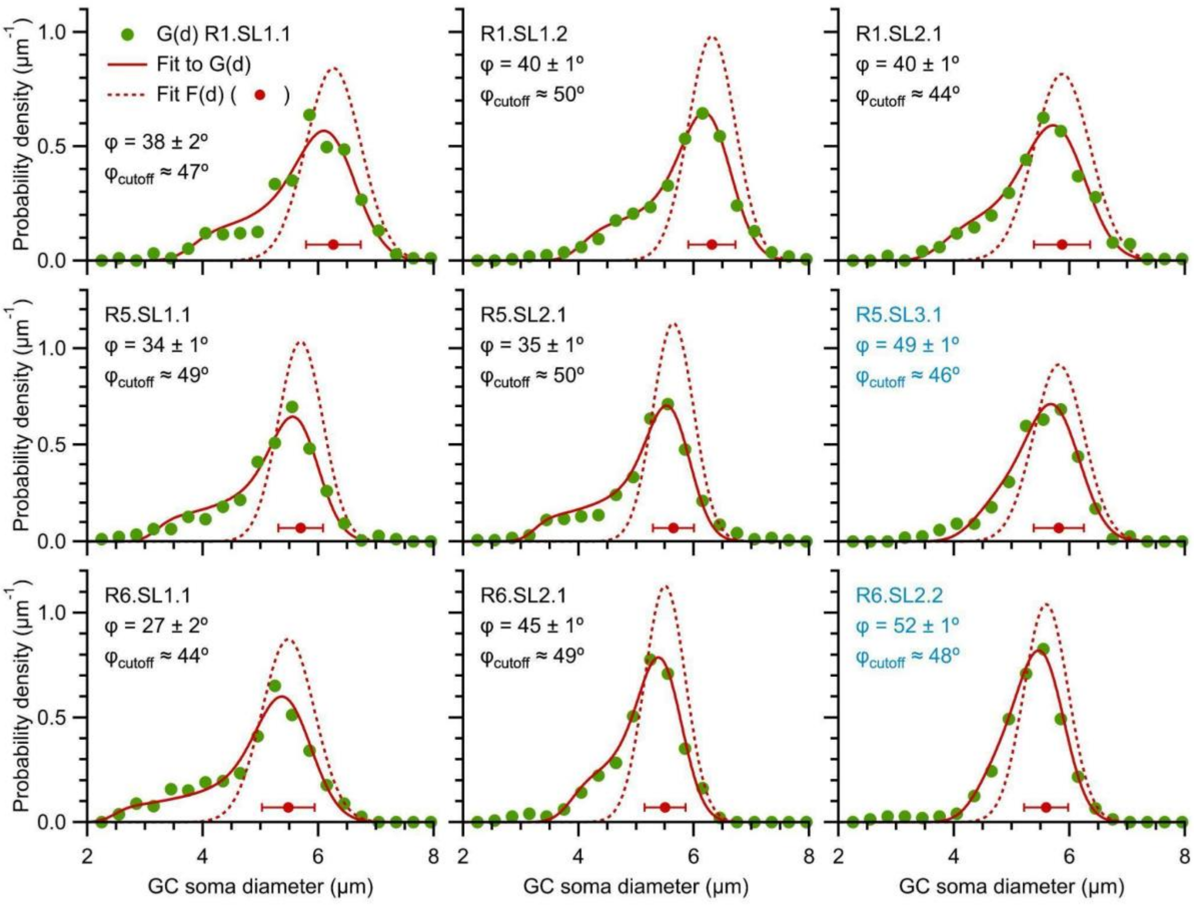

Fig S12. Keiding-model fits to G(d) of cerebellar GC somata.

G(d) (green circles) computed from outlines of GC somata (n = 494–638; 0.3 µm bins) measured from confocal images of the GC layer (Fig 2A1 and 2A2) where each row is for a different rat (R1, R5, R6) and each G(d) is computed from 1–3 images of a confocal z-stack. There were 2–3 tissue sections per rat (SL1, SL2, SL3). Each G(d) was curve fitted to Eq 1 (red solid lines) resulting in estimates for F(d) (red dotted lines and circles) and ϕ. Estimated ϕ_{cutoff} was computed via Eq 9. For all but two fits, estimated ϕ < estimated ϕ_{cutoff}. Comparisons of the diameter distributions within rats showed significant differences (KS test; $p < 0.05$), even after alignment on their mean μ_D.

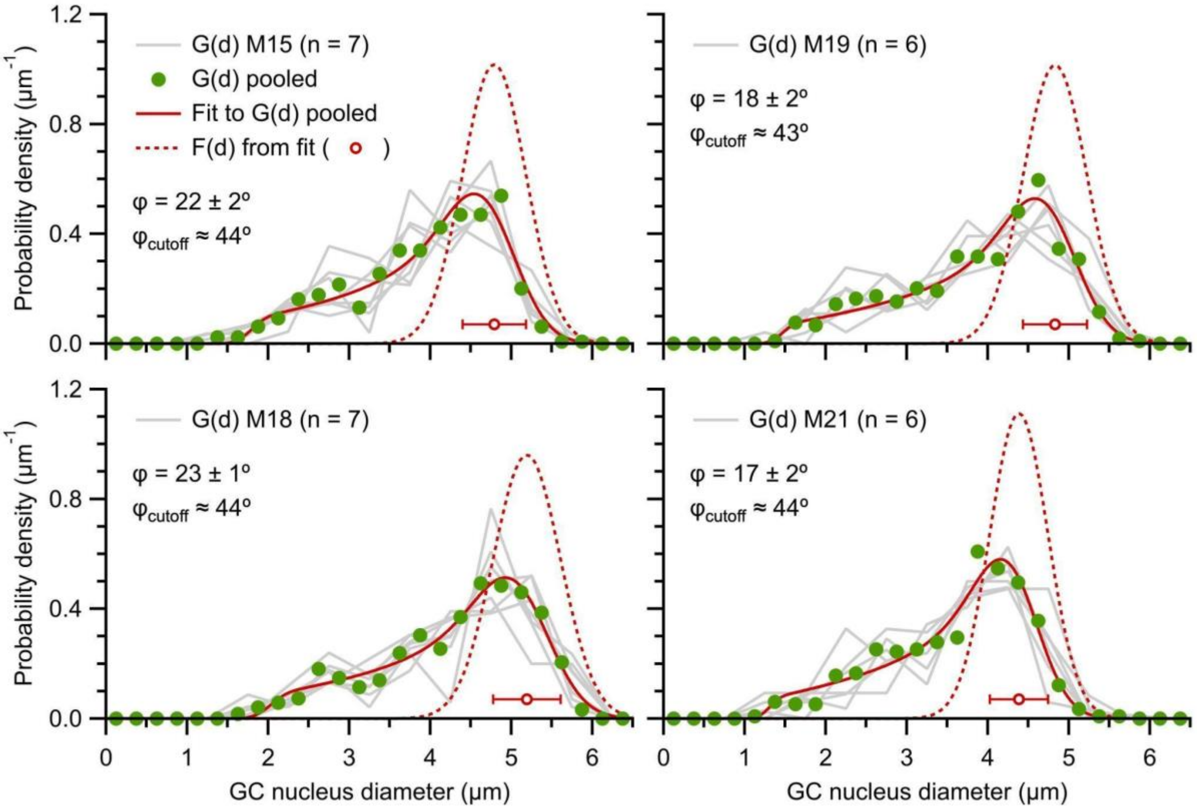

Fig S13. Keiding-model fits to G(d) of cerebellar GC nuclei.

G(d) (gray lines) computed from outlines of GC nuclei (n = 35–158 diameters; 0.5 μm bins) measured from TEM images of the GC layer (Fig 2B1 and 2B2) where each plot is for a different mouse (M15, M18, M19, M21). G(d) within mice (n = 6 or 7) were not significantly different and therefore pooled (green circles; n = 416–519 diameters; 0.25 μm bins). The resulting 4 pooled G(d) were curve fitted to Eq 1 (red solid lines) resulting in estimates for F(d) (red dotted lines and circles) and ϕ. Estimated ϕ_{cutoff} was computed via Eq 9. Image IDs: M15 = M15.L3.11, 12, 15, 22, 25, M15.L4.04, 08 (n=7); M18 = M18.N2.02, 05, 08, 51, M18.N3.17, M18.N4.10, 15 (n = 7); M19 = M19.O2.06, 12, 13, 20, 38, 44 (n = 6); M21 = M21.P5.16, 19, 33, 48, 52, 59 (n = 6).

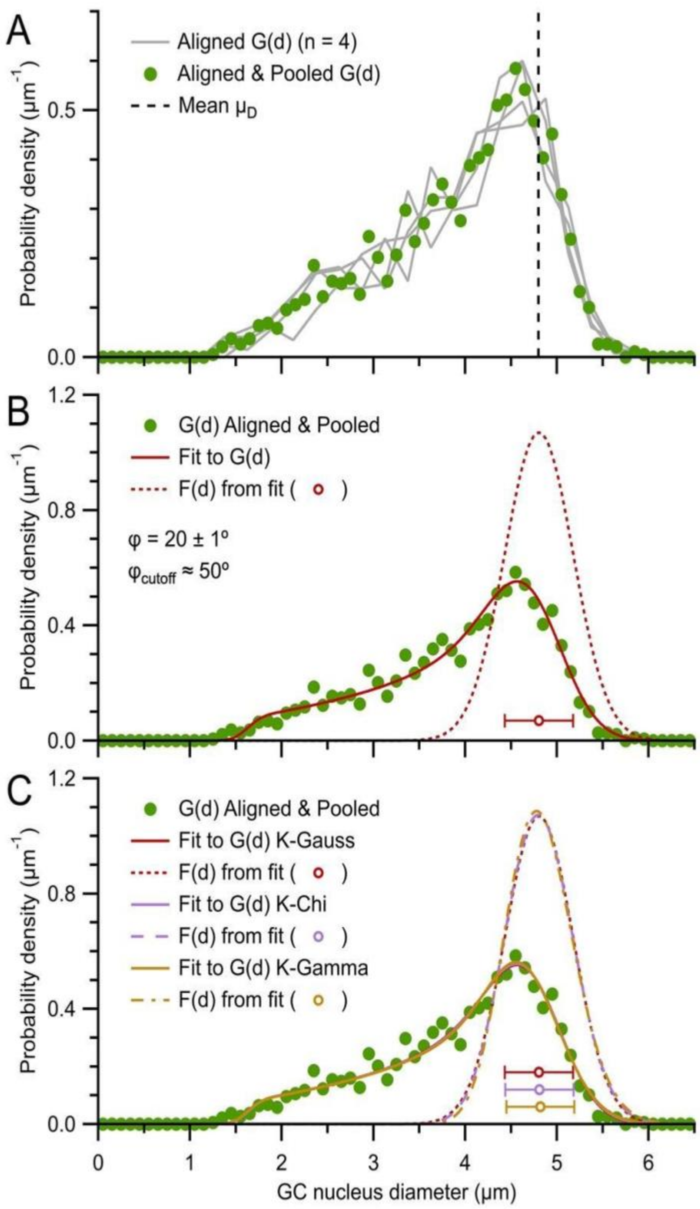

Fig S14. Analysis of aligned G(d) of cerebellar GC nuclei.

A. Pooled G(d) for each mouse in Fig S13 (gray lines) aligned at their mean μ_D (4.80 μm; black dashed line). After alignment, none of the 4 distributions were significantly different (KS test; $p > 0.16$) and were therefore pooled into a single G(d) (green circles; n = 1882 diameters; 0.1 μm bins). **B.** Aligned and pooled G(d) from **A** curve fitted to Eq 1 (red solid line; $\mu_D = 4.80 \pm 0.01$ μm, $\sigma_D = 0.37 \pm 0.01$ μm, $\phi = 20 \pm 1°$; estimated $\phi_{cutoff} = 50°$) with estimated F(d) (red dotted line and circle). **C.** The Keiding-model fit in **B**, which assumed a Gaussian function for F(d) (K-Gauss; Eq 5), compared to fits that assumed a chi distribution for F(d) (K-Chi; Eq 6; purple line; $f = 84.0 \pm 6.3$, $\beta = 0.28 \pm 0.02$ μm^2, $\phi = 20 \pm 1°$; $\mu_D \pm \sigma_D = 4.81 \pm 0.37$ μm) and gamma distribution for F(d) (K-Gamma; Eq 7; orange line; $f = 80.9 \pm 6.2$, $\beta = 0.041 \pm 0.003$ μm, $\phi = 20 \pm 1°$, d_0 fixed at 1.5 μm; $\mu_D \pm \sigma_D = 4.82 \pm 0.37$ μm). Comparison of F(d) from the fits show overlapping distributions (circles and error bars).

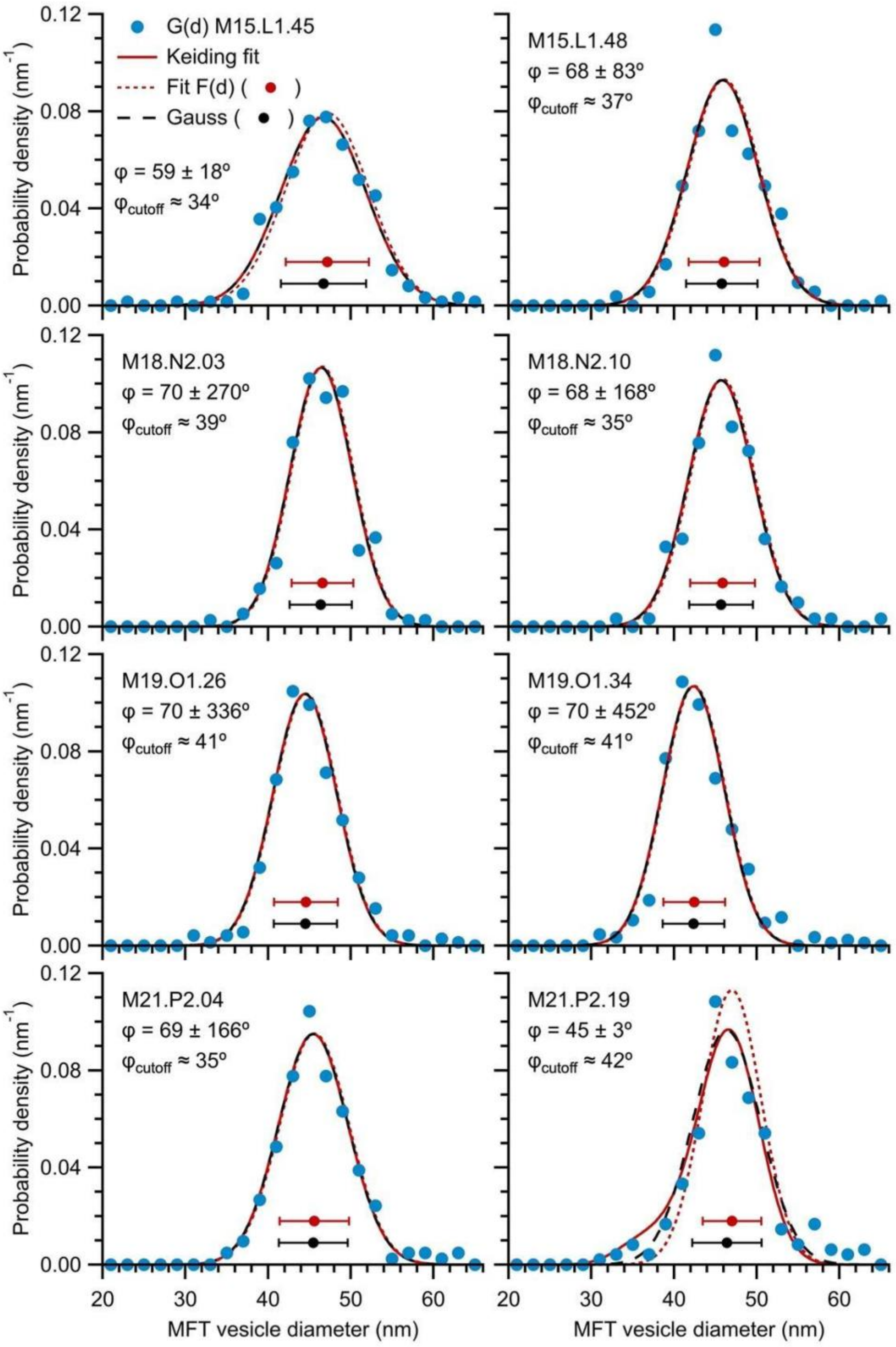

Fig S15. Keiding-model fits to G(d) of cerebellar MFT vesicles.

G(d) (blue circles) computed from outlines of MFT vesicles (n = 152–428; 2 nm bins) measured from TEM images of the GC layer (Fig 2C1 and 2C2) where each row is for a different mouse and each column is for a different MFT. Each G(d) was curve fitted to Eq 1 (red solid lines) resulting in estimates of F(d) (red dotted lines and circles). For all fits, ϕ has a large error and is greater than estimated ϕ_{cutoff} (Eq 9) indicating ϕ is indeterminable and G(d) ≈ F(d) (Fig 4C). Gaussian fits to the same G(d) (Eq 5; black dashed lines and circles) overlap the Keiding-model fits and estimated F(d).

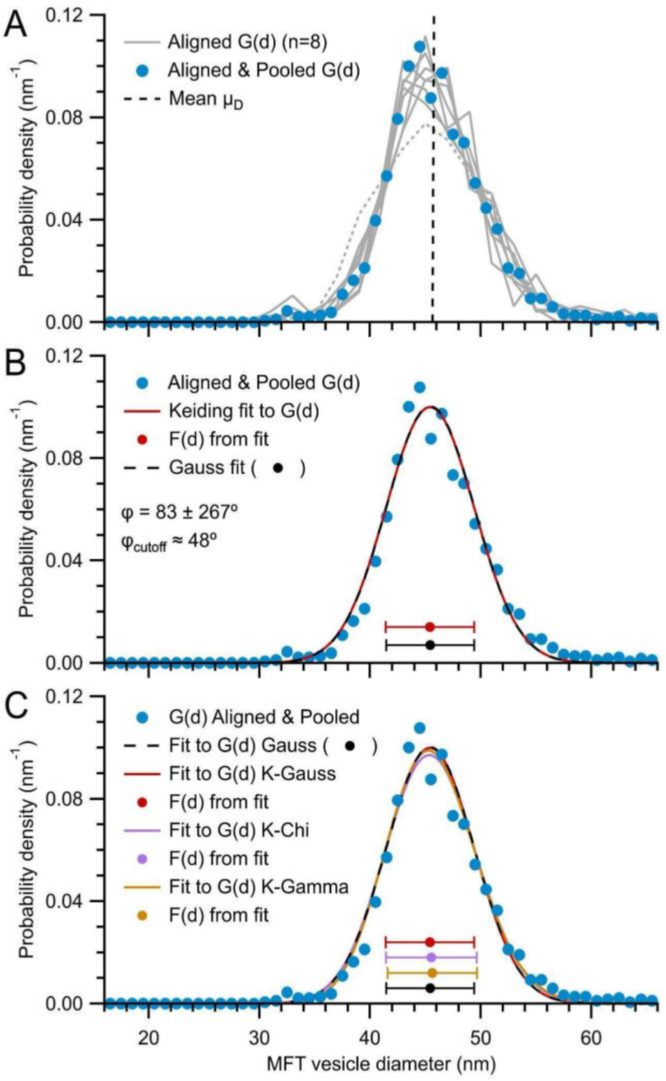

Fig S16. Analysis of aligned G(d) of cerebellar MFT vesicles.

A. G(d) of the 8 MFTs in Fig S15 (gray lines) aligned at their mean μ_D (45.7 nm; black dashed line). After alignment, only 2 of the 28 pairings of the distributions were significantly different (KS test; $p < 0.02$) both of which included the distribution with largest σ_D (gray dotted line; M15.L1.45). The aligned G(d) that were not significantly different (n = 7) were pooled into a single G(d) (blue circles; n = 1839 diameters; 1 nm bins). **B.** Aligned and pooled G(d) from **A** (blue circles) curve fitted to Eq 1 (red solid line; μ_D = 45.4 ± 0.1 nm, σ_D = 4.0 ± 0.2 nm, ϕ = 83 ± 267°) with estimated F(d) (red circle and error bars) and ϕ_{cutoff}. As in Fig S15, ϕ is indeterminable and G(d) ≈ F(d). A Gaussian fit to G(d) (Eq 5; black dashed line and circle; μ_D = 45.4 ± 0.1 nm, σ_D = 4.0 ± 0.1 nm) overlaps the Keiding-model fit and estimated F(d). **C.** The Keiding-model fit in **B**, which assumed a Gaussian function for F(d) (K-Gauss; Eq 5), compared to fits that assumed a chi distribution for F(d) (K-Chi; Eq 6; purple line; f = 61.8 ± 1.9, β = 33.9 ± 1.0 nm^2, ϕ = 90 ± 49°; $\mu_D \pm \sigma_D$ = 45.5 ± 4.1 nm) and gamma distribution for F(d) (K-Gamma; Eq 7; orange line; f = 127.0 ± 3.3, β = 0.36 ± 0.01 nm, ϕ = 89 ± 137°, d_0 fixed at 0 nm; $\mu_D \pm \sigma_D$ = 45.6 ± 4.0 nm). Comparison of F(d) from the fits shows overlapping distributions (circles and error bars).

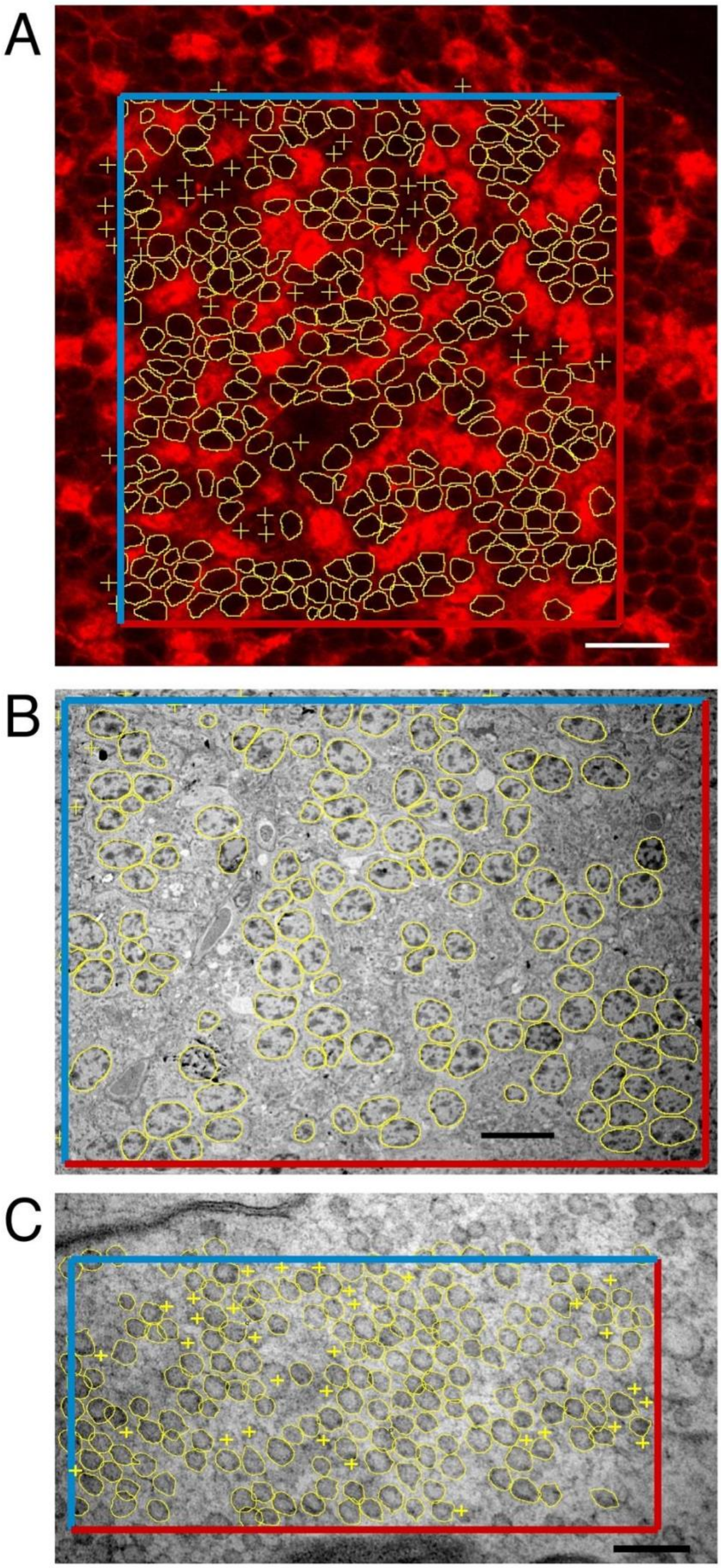
A
B
C

Fig S17. Estimating the 2D density of GC somata and nuclei and MFT vesicles from 2D images.

A. To compute λ_{2D} of GC somata of rats in a confocal image, a rectangular ROI (119.0 × 126.3 μm) was drawn within the GC layer and two adjacent borders were designated as inclusive and the other two as exclusive (blue and red). Somata were counted if they touched the inclusive borders or were completely contained within the ROI; they were not counted if they touched the exclusive borders. λ_{2D} = 21,354 mm^{-2}, computed as count (321) per ROI area (15032 μm^{2}). Somata were outlined if they were well delineated, otherwise they were marked via crosses (yellow). Scale bar 20 μm. Image ID R5.SL2.1.

B. Same as **A** for GC nuclei of mice in a TEM image. ROI = 88.0 × 65.3 μm. n = 130 nuclei. λ_{2D} = 22,619 mm^{-2}. Scale bar 10 μm. Image ID M18.N2.51.

C. Same as **A** for MFT vesicles of mice in a TEM image. ROI = 1147 × 561 nm. n = 209 vesicles. λ_{2D} = 324.5 μm^{-2}. Scale bar 150 nm. Image ID M15.L1.48.

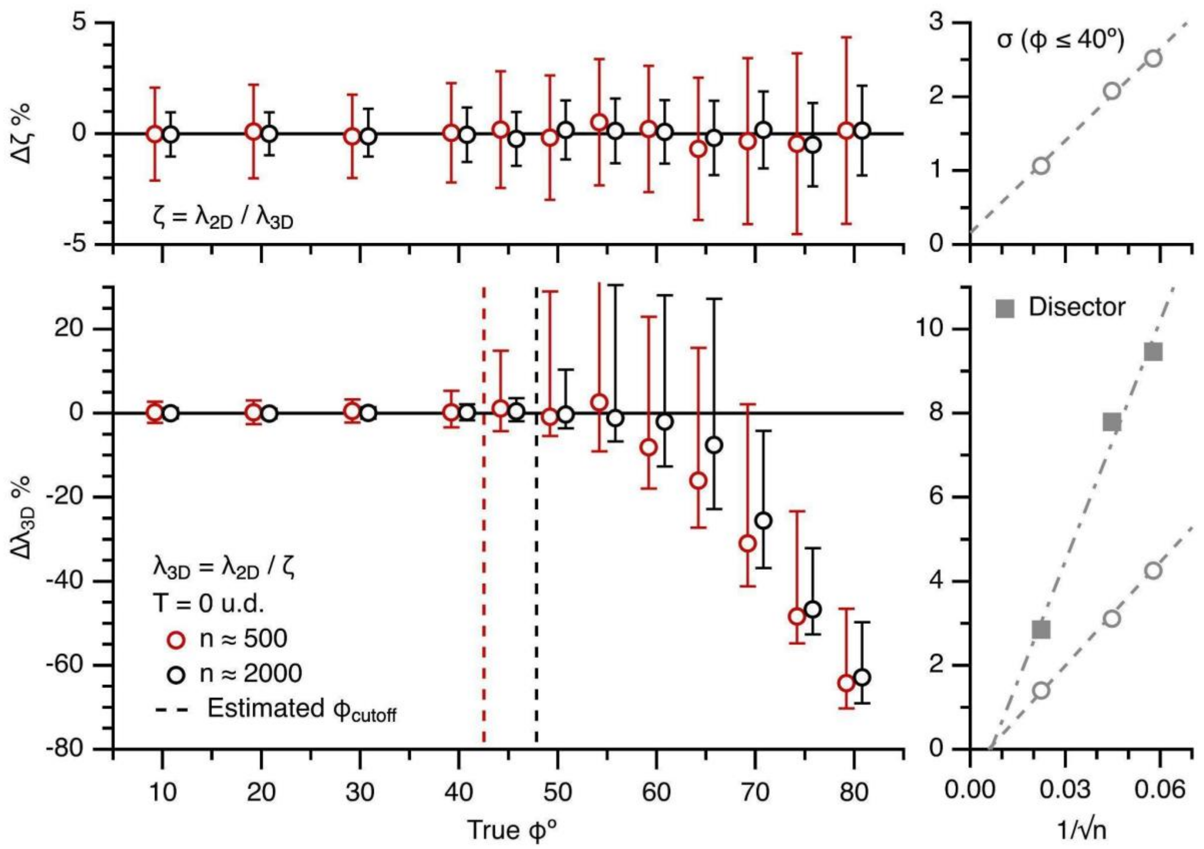

Fig S18. The Keiding model accurately estimates λ_{3D} from λ_{2D} for true ϕ < estimated ϕ_{cutoff} (500 vs. 2000 simulated diameters).

Left column: average $\Delta\zeta$ (top) and $\Delta\lambda_{3D}$ (bottom) vs. true ϕ for the simulations in Fig S4, computed from ~500 and ~2000 diameters (red and black circles) for T = 0 u.d. as described in Fig 10. Dashed lines denote estimated ϕ_{cutoff} (~43 and 48°). Data x-scales shifted ±0.8° to avoid overlap.

Right column: 68% confidence interval (σ_{Δ}) of $\Delta\zeta$ and $\Delta\lambda_{3D}$ (open circles; averaged across true ϕ = 10–40°) vs. 1/√n (n = 300, 500 and 2000 diameters) curve fitted to a linear function (dashed lines). Data from disector simulations with no added bias is shown for comparison (solid squares; Fig 12, ϕ_{bias} = 0°).

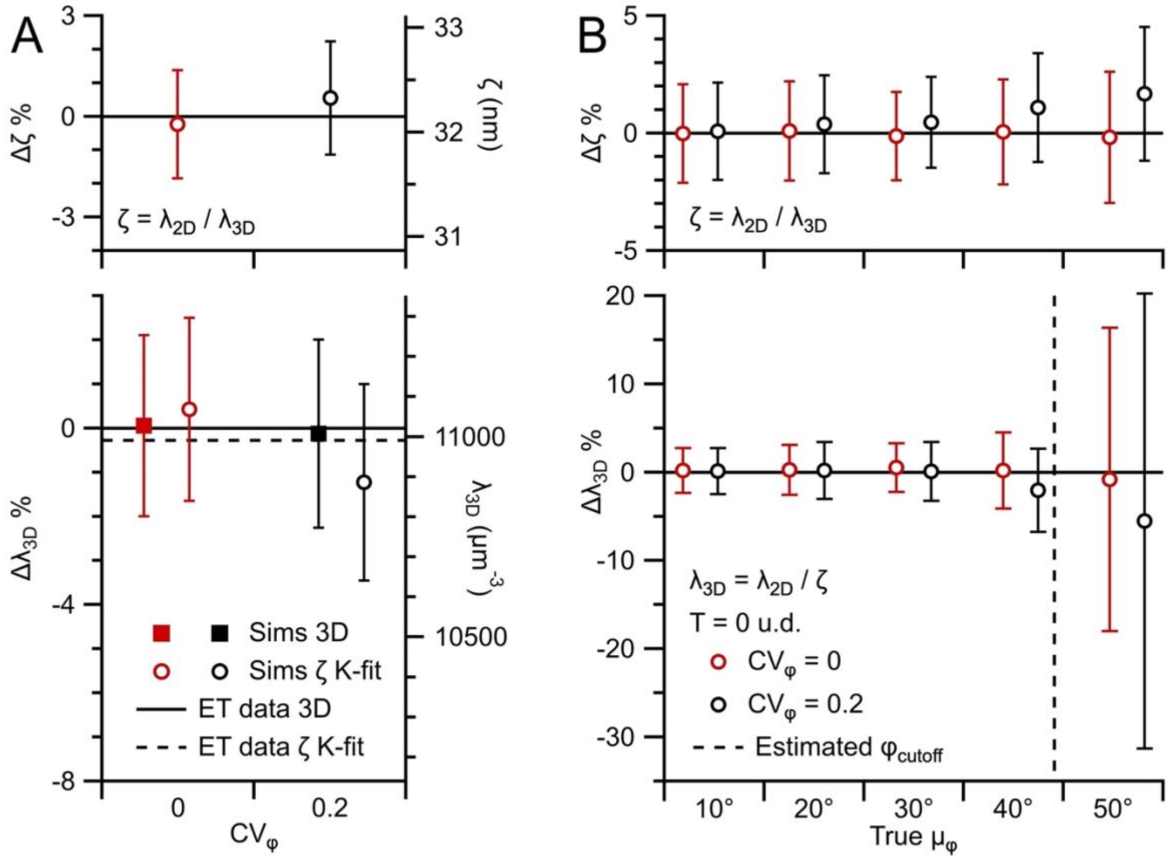

Fig S19. The Keiding-model fixed-ϕ assumption introduces only small errors when estimating λ_{3D} from λ_{2D}.

A. Average Δζ (top) and $\Delta\lambda_{3D}$ (bottom) for simulations of the z-stack analysis in Fig 11A2 (ET11) where lost caps are defined by a fixed-ϕ or Gaussian-ϕ model (CV_ϕ = 0 or 0.2; red vs. black symbols) and the scanning xy-plane (T = 0 nm) was z-shifted 261 times in 0.5 nm steps resulting in ~10,500 2D diameters. Δζ and $\Delta\lambda_{3D}$ were computed as described in Fig 10 (open circles; true ϕ = μ_ϕ). For the volumetric analysis, estimated $\lambda_{3D} = N_{3D}/(Area_{xy} \cdot \zeta)$ where ζ was computed via μ_D and μ_ϕ of those vesicles sampled by the z-stack (3D; $N_{3D} \approx 200$; closed squares). Simulation true λ_{3D} = 10,825 µm^{-3} (VF = 0.45), μ_D = 42.7 nm, μ_ϕ = 42.0°, $Area_{xy}$ = 0.117 µm^2. Equivalent ζ and λ_{3D} for the ET z-stack analysis of MFT vesicles (ET11) is shown for comparison (black solid and dashed lines) which are consistent with the Gaussian-ϕ simulations. Estimated $\phi_{cutoff} \approx 51°$ (Eq 9).

B. Average Δζ (top) and $\Delta\lambda_{3D}$ (bottom), for the fix-ϕ and Gaussian-ϕ simulations in Fig S11 (CV_ϕ = 0 or 0.2; red vs. black) and T = 0. The small positive biases in Δζ for the Gaussian-ϕ simulations indicate estimated ζ is marginally too large (i.e. Eq 2 is only approximately correct for Gaussian-ϕ conditions); however, these biases created negligible biases in λ_{3D} for $\mu_\phi < 50°$. Estimated $\phi_{cutoff} \approx 43°$ (Eq 9). Fixed-ϕ data is from Fig 10.

References

Abercrombie M. Estimation of nuclear population from microtome sections. Anat Rec. 1946 Feb;94: 239–47. doi: 10.1002/ar.1090940210

Cruz-Orive LM. Distribution-free estimation of sphere size distributions from slabs showing overprojection and truncation, with a review of previous methods. J Microsc. 1983 Sep;131(3): 265–290. doi: 10.1111/j.1365-2818.1983.tb04255.x

Floderus S. Untersuchung üeber den Bau der menschlichen Hypophyse mit besonderer Berüecksichitigung der quantitative mikromorphologischen Verhäeltnisse. Acta Path Microbiol Scand. 1944;53: 1–276.

Keiding N, Jensen ST, Ranek L. Maximum likelihood estimation of the size distribution of liver cell nuclei from the observed distribution in a plane section. Biometrics. 1972 Sep;28(3): 813–829. doi: 10.2307/2528765

Konigsmark BW. Methods for the counting of neurons. In: Nauta WJH, Ebbesson SOE, editors. Contemporary Research Methods in Neuroanatomy. Berlin, Heidelberg: Springer Berlin Heidelberg; 1970. pp. 315–340. doi: 10.1007/978-3-642-85986-1_14

Luther PK, Lawrence MC, Crowther RA. A method for monitoring the collapse of plastic sections as a function of electron dose. Ultramicroscopy. 1988;24(1): 7–18. doi: 10.1016/0304-3991(88)90322-1

Nguyen TM, Thomas LA, Rhoades JL, Ricchi I, Yuan XC, Sheridan A, et al. Structured cerebellar connectivity supports resilient pattern separation. Nature. 2023 Jan 19; 613(7944):543-549. doi: 10.1038/s41586-022-05471-w

Rothman JS, Kocsis L, Herzog E, Nusser Z, Silver RA. Physical determinants of vesicle mobility and supply at a central synapse. eLife. 2016 Aug 19;5: e15133. doi: 10.7554/eLife.15133

Weibel ER, Paumgartner D. Integrated stereological and biochemical studies on hepatocytic membranes. II. Correction of section thickness effect on volume and surface density estimates. J Cell Biol. 1978 May;77(2): 584–97. doi: 10.1083/jcb.77.2.584

Wicksell SD. The corpuscle problem: a mathematical study of a biometric problem. Biometrika. 1925 Jun;17(1/2): 84–99. doi: 10.2307/2332027
